# Manipulation Cases in Free Will and Moral Responsibility, Part 1: Cases and Arguments

**DOI:** 10.1111/phc3.70009

**Published:** 2024-11-27

**Authors:** Gabriel De Marco, Taylor W. Cyr

**Affiliations:** ^1^ Faculty of Philosophy University of Oxford Uehiro Oxford Institute Oxford UK; ^2^ Department of Classics and Philosophy Samford University Birmingham Alabama USA

**Keywords:** compatibilism, free will, manipulation, moral responsibility

## Abstract

A common style of argument in the literature on free will and moral responsibility is the Manipulation Argument. These tend to begin with a case of an agent in a deterministic universe who is manipulated, say, via brain surgery, into performing some action. Intuitively, this agent is not responsible for that action. Yet, since there is no relevant difference, with respect to whether an agent is responsible, between the manipulated agent and a typical agent in a deterministic universe, responsibility is not compatible with the truth of determinism. This paper introduces some key types of manipulation cases, the schema for a manipulation argument against compatibilism, the hard‐line/soft‐line categories of responses to manipulation arguments, and various issues that have become important in discussions of manipulation cases and arguments.

## 1 Introduction

Cases of manipulation and arguments that make reference to them are now pervasive in the philosophical literature on free will (or free action) and moral responsibility (hereafter “responsibility” for short). One of the central debates in that literature concerns whether freedom and responsibility are compatible with causal determinism. If a universe is deterministic, then, given the past and the laws of nature, there is only one possible future. As their names suggest, incompatibilists take freedom and responsibility to be incompatible with determinism, whereas compatibilists take them to be compatible. And one of the main arguments for incompatibilism in the contemporary debate is the manipulation argument against compatibilism, which by now exists in several variations that feature different cases of manipulation.^1^

This paper introduces some key types of manipulation cases, the schema for a manipulation argument against compatibilism, the hard‐line/soft‐line categories of responses to manipulation arguments, and various issues that have become important in discussions of manipulation cases and arguments. The two sequels to this paper summarize the debate concerning the two main types of soft‐line views.

## 2 Manipulation Cases

We can begin by introducing some of the classic cases of manipulated agents. Such cases are set in deterministic universes and feature one agent, which we will sometimes refer to as Manipulator, performing some intervention on another, which we will sometimes refer to as Victim, in order to get Victims to perform some action. Consider the first case that appears in Pereboom's widely discussed Four Case Argument against compatibilism:

*Plum*: A team of neuroscientists has the ability to manipulate Professor Plum’s neural states at any time by remote optogenetic neural stimulation. In this particular case, they do so by pressing a button just before he begins to reason about his situation, which they know will produce in him a neural state that realizes a strongly egoistic reasoning process, which they know will deterministically result in his decision to kill White. Plum would not have killed White had the neuroscientists not intervened, since his reasoning would then not have been sufficiently egoistic to produce this decision.

(Fischer et al. 2024, 98)

It seems that Plum is not morally responsible for deciding to kill White, or for the killing itself, despite the fact that he is stipulated to satisfy various compatibilist conditions on responsibility^2^; for example, his action issues from a process that is appropriately responsive to reasons, and conforms to higher‐order attitudes.

Now consider a pair of cases from Mele:

*Chuck*: Chuck enjoys killing people…When he was much younger, Chuck enjoyed torturing animals, but he was not wholeheartedly behind this….He freely set out to ensure that he would be wholeheartedly behind his torturing of animals and related activities, including his merciless bullying of vulnerable people, and he was morally responsible for so doing…His strategy worked. Today, he stalked and killed a homeless man, Don

(2019: 19–20)

*Sweet Sally*: When [Sweet] Sally crawled into bed last night, she was one of the kindest, gentlest people on Earth…Sally’s character was such that intentionally doing anyone serious bodily harm definitely was not an option for her: Her character—or collection of values—left no place for a desire to do such a thing to take root…But Sally awakes with a desire to stalk and kill a neighbor, George. Although she had always found George unpleasant, she is very surprised by this desire. What happened is that, while Sally slept, a team of psychologists that had discovered the system of values that make Chuck tick implanted those values in Sally after erasing her competing values. They did this while leaving her memory intact, which helps account for her surprise. Sally reflects on her new desire. Among other things, she judges, rightly, that it is utterly in line with her system of values…Seeing nothing that she regards as a good reason to refrain from stalking and killing George, provided that she can get away with it, Sally devises a plan for killing him; and she executes it—and him—that afternoon…

(2019: 20–1)

Although Sweet Sally and Chuck have similar motivations and values relevant to the decision at the time of their respective killings, and although they may have similar abilities or capacities to recognize and respond to reasons, or to deliberate on the basis of informed deliberation, many will judge that, while Chuck is morally responsible for killing Don, Sally is not morally responsible for doing so.^3^

*Plum* and *Sweet Sally* are cases of *mid‐life* manipulation. These can be contrasted with cases of what we will call *original design* manipulation, such as the following, again drawn from Mele:

*Ernie*: Diana, a goddess with special powers and knowledge, wants event E to occur 30 years after time t. Diana knows what the state of the universe is just before t and she knows the laws of nature of her deterministic universe. With these things in mind, she creates a zygote, Z, in Mary at time t which will develop into Ernie. Diana does this knowing that, given the state of the world and the laws, Ernie will, 30 years in the future, perform action A which will bring about event E.

(2006: 188–9)^4^

Like the cases of Plum and Sweet Sally above, many will think that Ernie is not responsible for A‐ing. Yet unlike the two cases above in which the manipulator intervenes on an already‐existing, full‐fledged agent, Diana does all of her work before Ernie is even born.^5^

## 3 The Manipulation Argument Against Compatibilism

There are variations, but many instances of the manipulation argument against compatibilism begin by presenting a case of manipulation—like one of those above—and then provide some reasoning along the lines of the following:

1. The manipulated agent (i.e., Victim) is neither free nor responsible for the relevant action. (*No‐FR*)
2. There is no freedom or responsibility‐relevant difference between Victim and ordinary (non‐manipulated) agents in deterministic worlds. (*No‐Diff*)
3. Therefore, ordinary agents in deterministic worlds are neither free nor responsible for their actions (i.e., compatibilism is false).

If one thinks that the Victims above—Plum, Sally, or Ernie—do not act freely and are not responsible, then one would agree with the claim in the first premise, which we refer to as *No‐FR.* ^6^ Though Premise 2 comes in various forms, the rough idea is that, with respect to features relevant to whether an agent is free or responsible, there is no relevant difference between Victim and a standard agent in a deterministic universe.^7^ We will refer to the claim in the second premise as *No‐Diff.*

The conclusion of the argument can also vary. A simple version of the conclusion is stated above, concluding that compatibilism, so understood, is false. Yet sometimes, such arguments may conclude with a stronger claim, further suggesting, roughly, that a world's being deterministic somehow explains why compatibilism is false. In order to achieve this conclusion, however, the argument will need to be supplemented with some further premise(s).^8^ Alternatively, one could go weaker, arguing simply that standard, or perhaps existing, compatibilist views are false.^9^ Going weaker still, one could use such cases to argue against specific compatibilist views—e.g., Frankfurt's view (1971)—though not all.^10^ And, as we'll see later, such cases can be used against some libertarian views as well; that is, incompatibilist views which *also* hold that we sometimes act freely or responsibly.

A brief note on presentation. What is at issue in this discussion is *directly* free action and *direct* responsibility, not derivative freedom or responsibility; for example, responsibility one has for an action in virtue of being responsible for some *other* item, say, a previous action. Since agents' fulfillment of the epistemic condition on direct responsibility is not typically in question in these debates, the idea is that if Victim is not responsible, it is in virtue of their failure to meet the freedom condition on responsibility. For the sake of simplicity, we will thus only talk about agents' responsibility and not about free action or free will, though many of the works cited here use the latter terminology.^11^

## 4 The Dialectic: Soft Versus Hard‐Line Responses

We can begin by describing two main categories of responses to the argument: hard‐line and soft‐line (McKenna 2008, 143).^12^ Hard‐line responses to the manipulation argument deny *No‐FR*; they accept that Victim is responsible for the relevant action. These are the topic of the next section.

Soft‐line responses object to *No‐Diff*, and are the subject of the two sequels to this article. Such responses tend to offer conditions on direct responsibility and argue that, whereas standard agents meet the proposed condition, Victims in manipulation cases do not.^13^ One type of soft‐line response involves a *manipulator‐focused view—*a view on which, roughly, the reason that Victim lacks responsibility (or lacks full responsibility) is because of the way the action is related to the Manipulator (see Part 2).^14^ Another type of soft‐line response invokes a *bypassing view*—on which something about the way the Victim's capacities were bypassed undermines (or mitigates) their responsibility (see Part 3).^15^

Though this terminology can be useful, it is worth bearing in mind that not all responses fit neatly into this division (see Sections 6 and 7). Further, one can take a hard‐line response with respect to some cases of manipulation while taking a soft‐line response with respect to others.^16^ In fact, as we will see in Parts 2 and 3, proponents of soft‐line responses to some cases often explicitly take the hard‐line with respect to others. Thus, whether a response, or a view, constitutes a soft‐ or hard‐line will be relative to the case under consideration. However, here it is worth mentioning a worry—sometimes described as the *main* worry—with soft‐line replies in general.^17^

A soft‐line respondent can offer a necessary condition on responsibility that a particular victim fails to meet, and thus respond to a *particular* case. Yet for any soft‐line view one offers, the thought goes, it is plausible that the proponent of a manipulation argument will simply be able to produce a new case in which Victim *does* meet the proposed necessary condition. For example, consider again the case of Ernie. Given that Diana, the manipulator, does all of her work before he is even born, bypassing views cannot account for a difference between Ernie and a standard agent in a deterministic universe since there is no difference in terms of whether their capacities, once developed, were bypassed.

## 5 Hard Lines

The “hardest” type of hard‐line response maintains that manipulation (even mid‐life) does not affect even the degree of Victim's responsibility. For example, Frankfurt says:

When a person chooses to act in order to acquire a benefit or in order to escape an injury, the degree to which his choice is autonomous and the degree to which he acts freely do not depend on the origin of the conditions which lead him to choose and to act as he does.

(1988, 45–6)

On Frankfurt's view, it is only what is going on “in the agent's head” (at or around the time of action) that matters for their freedom and responsibility, and so he is committed to taking a hard‐line response to nearly all standard cases of manipulation.^18^

Insofar as one finds it intuitive that manipulated agents are not responsible, taking the hard‐line approach will involve seemingly biting a bullet. However, there are now several proposals for how the blow might be softened while maintaining a hard‐line approach.

Some authors suggest that our intuitions about Victims are really tracking something other than Victim's responsibility. For instance, Fischer (2017) argues that our intuitions are tracking the victim's autonomy, and that autonomy is not necessary for responsibility.

Alternatively, one might worry that insofar as we think Manipulator is blameworthy for Victim's action, we are mistakenly inferring that Victim is not. More broadly, some argue that our reaction to Manipulator might be somehow throwing off our reaction to Victim.^19^ In response, one could modify the case, such that the manipulator fails to meet standard conditions on responsibility; for example, in the case of Ernie above, one could stipulate that Diana is “stark raving mad”, and thus not responsible (Mele 2006, 198, *n*. 16). One might also go further and remove Manipulator altogether. Consider a *natural force* case, modeled on the case of Sally above:

*Natalie:* Natalie was just as sweet as Sally was before her transformation. However, due to a strange electro‐magnetic storm over her house while Natalie slept last night, she underwent a change much like Sally’s. She wakes up, deliberates in a similar fashion, decides to kill her neighbor, and does so.^20^

If one thinks Natalie lacks responsibility, it is unlikely that this will be due to our reaction to Manipulator, since there is none in this case.^21^

Another suggestion reverses the order of things. Rather than taking our judgments about manipulated agents to show us something about ordinary agents, we take our judgments (or lack thereof) of ordinary agents in deterministic universes to show us something about manipulated agents. McKenna initially developed this line in response to Pereboom's Four Case Argument.^22^ As noted above, *Plum* is the first of Pereboom's cases, and his second is similar to the case of *Ernie* (where Plum is pre‐programmed to behave in the way that his manipulators intend). In the third case, Plum grows up in an egoistic society that instills in him the very same tendencies as he is given in the earlier cases, and with the same result. Finally, the fourth case is an ordinary deterministic world in which Plum comes to have the same tendencies and to perform the same action without any manipulators in the picture. Pereboom begins with Case 1 and attempts to carry the *No‐FR* judgment over to Case 4 by arguing that there is no relevant difference between any two adjacent cases.^23^ McKenna retorts that we should start with Case 4, where an open‐minded inquirer would not have decisive reason to deny the agent's responsibility, and so the inquirer should be agnostic about the agent's responsibility. Then, using the very same no‐relevant‐difference claims as Pereboom accepts, McKenna argues that the inquirer should come to agnosticism about Victim's responsibility in Case 1.^24^

Often this reversal strategy is supplemented by an appeal to real‐life cases in which people undergo radical changes, without foreseeing or controlling these changes; like the experience some new parents or religious converts report.^25^ McKenna says that such cases “are the closest a compatibilist can come to providing a positive intuitive basis for her thesis regarding the possibility of manipulated agents” (2008, 156–7) and suggests that we can explain the difference in our intuitions about Case 1 and Case 4 by attending to the fact that “[o]ur intuitions have evolved along with our ordinary practices” and so will be “indecisive” when “tested in extremely different contexts” (2008, 157).^26^

Yet another suggestion focuses on subjects of mid‐life manipulation, and claims that, even though they are responsible with respect to the relevant action, there remains a relevant difference between Victim and a standard agent.^27^ For example, McKenna (2004, 183) claims that victims of mid‐life manipulation are just as responsible for their post‐manipulation behavior as similar non‐manipulated agents, but manipulated agents are responsible for fewer things, since non‐manipulated agents, but not the victims of manipulation, can be responsible for the characters from which they act.

Relatedly, one could instead claim that, while Victim is responsible, they are responsible for the relevant behavior to a lesser degree than their non‐manipulated counterparts. For example, Cyr 2019, 2020a) argues that compatibilists must take this sort of approach to certain cases of mid‐life manipulation. And Montminy and Tinney (2018) respond to Pereboom's manipulation argument by arguing that Plum in Case 1 has mitigated blameworthiness in virtue of the fact that it is harder for him—compared to a standard agent—to do the right thing since “only in a relatively small proportion of nearby worlds in which he has an opportunity to kill someone for personal gain, Plum1 refrains from doing so” (2018, 280).^28^

Another response appeals to a particular view of responsibility, on which it requires, roughly, the ability to act in the right way and for the right reasons.^29^ Yet in many manipulation cases, Victim is stipulated to lack this ability, or plausibly does. This view does not face counterexamples from such cases. There are, however, other variations. Suppose that, rather than make Sweet Sally more like Chuck, Manipulators instead make Chuck more like Sweet Sally, who goes on to do a variety of charitable deeds that would have been unthinkable to him before the manipulation (Mele 2019, 20–24). If one thinks that Chuck is praiseworthy for these deeds—as some proponents of such theories do (Nelkin 2011, 56–60; Whittle 2021, 169)—then the case serves to further support the view. Yet many will judge Chuck not to deserve any credit, just as Sweet Sally seems not to deserve any blame, and these asymmetry views will not be able to account for such a judgment about Chuck.^30^

## 6 Parallel Cases

A different type of response to manipulation arguments against compatibilism is to point out that determinism is not necessary for manipulation cases to elicit the intuition that Victim lacks moral responsibility. Consider any of the standard cases above yet suppose that very shortly before the relevant decision, there is a small indeterministic chance that the agent will, instead, become unconscious (Kearns 2012; Mele 2005, 2006, 142).^31^ Victim still intuitively lacks responsibility, yet is not determined to act as she does.^32^ Moreover, there are more robust versions of such cases, ones in which Victim meets standard *in*compatibilist conditions on responsibility; for example, the agent could have done otherwise, holding the past and the laws fixed. Call such cases *parallel cases.* There are a variety of parallel cases on offer, and they are used to argue for different things.^33^ But consider the following, which is a slight variation on a parallel case offered by Mele (2019, 124–5)^34^:

*Paru:* Paru is just as sweet as Sally was before she was manipulated. However, Paru dislikes her twin neighbors, Peter and Pecker. She is manipulated in much the same way as Sally was. The next day, just like Sally, she considers whether to kill her neighbor. However, given that she is in a world where indeterminism shows up in just the right place, it is open, given the past and the laws, and up until the requisite time, whether she will choose to kill Peter, choose to kill Pecker, or simply become unconscious. She decides to kill Peter and does so.

Paru is a parallel case of mid‐life manipulation. If one agrees that Paru is not responsible for killing Peter, then one might think that manipulation cases can pose a problem for at least some libertarians as well as well as compatibilists.^35^ From this, one might conclude that such libertarians have lost their dialectical advantage over compatibilists.^36^ Alternatively, one might think this serves to improve the prospects of the responsibility skeptic.^37^

Here it is worth mentioning that though there are detailed versions of parallel mid‐life cases—like *Paru* above—there is only one such case, that we know of, that is of the original design variety (Cyr 2020b, 64–5).^38^ Yet it is difficult to produce a version of this case in which Victim both a) meets standard libertarian conditions, and b) intuitively lacks responsibility. Without such a case, parallel cases may only even the scoreboard with respect to cases of the mid‐life variety.^39^ We now turn to a series of dilemmas offered as responses to manipulation arguments.

## 7 The Dilemmas

Making use of parallel cases, King (2013) argues that a libertarian who offers a manipulation argument will need to offer some further argument for *No‐FR* which can apply to both standard and parallel versions of the argument.^40^ If the libertarian appeals to some feature unique to the manipulation, then *No‐Diff* will come out false. Alternatively, one could appeal to some sort of sourcehood condition—on which responsibility for an action requires that an agent is its “source.” However, in order for such an argument to be successful against compatibilism, the condition must be such that Victim's failure to be a source generalizes to other agents in deterministic universes, yet does not generalize to all agents in indeterministic universes. In doing this, however, the libertarian would be appealing to a contentious incompatibilist sourcehood thesis, which would be dialectically infelicitous.

Kearns offers a related dilemma. Either the manipulation undermines Victim's responsibility, or it does not (2012: 388–9). If the manipulation *does* undermine the agent's responsibility, then it looks like there is a relevant difference between the manipulated agent and a standard agent in a deterministic universe. If so, then *No‐Diff* is false, and a compatibilist can accept the claim that Victim lacks responsibility without thereby committing to the claim that standard agents in deterministic universes also lack it. If the manipulation does *not* undermine the agent's responsibility, then manipulation does not play an essential role in the argument, and it should be possible to give other cases of determined agents who intuitively lack responsibility but are not manipulated. Kearns then offers various cases which do not elicit this intuition (387).^41^

And Vihvelin offers a dilemma concerning our intuitions, on the supposition that we have the intuition underlying *No‐FR* because we can see that Victim is the tool of, or controlled by, Manipulator.^42^ The idea is that either the explanation of this intuition—that Victim is a tool of Manipulator—*justifies* the intuition, or it does not. If it does, then there is a relevant difference between Victim and a standard agent. If it does not, then the intuition does not have evidential value.^43^

## 8 Todd's Variations

Finally, Todd has developed two variations on manipulation arguments which appeal to different judgments concerning Victim. First is Todd's “Modified Manipulation Argument” (MMA). The basic idea is that proponents of standard manipulation arguments against compatibilism take on too heavy a burden by relying on the claim that Victim *fully* lacks responsibility for the relevant action. According to Todd (2011), all they really need to rely on is the claim that the blameworthiness of manipulated agents is *mitigated*. Here is the MMA^44^:

1. If blameworthiness is mitigated for Ernie, blameworthiness is mitigated if mere causal determinism is true.
2. If blameworthiness is mitigated if mere causal determinism is true, then compatibilism is false.
3. Blameworthiness is mitigated for Ernie.
4. Compatibilism is false.

Someone who thinks that Ernie is not responsible *at all* will accept (3); yet someone who is not convinced of this claim, and thereby is not convinced of *No‐FR* in the standard argument, might still accept (3). Thus, the MMA can be used to convince such people, and change the dialectical situation the compatibilist faces.

There are various responses available to the compatibilist. First, since (1) is a version of *No‐Diff* (with respect to Ernie), a particular sort of soft‐line theorist who denies that premise with respect to Ernie in the standard version might deny (1) of the MMA as well. Second, one could take a hard‐line with respect to this case, and deny (3). Third, one might deny (2). Capes (2013) and Tierney (2013) both argue that compatibilists can accept the view that determinism mitigates responsibility without giving up compatibilism.^45^

Todd's other approach concerns the standing to blame (2012).^46^ In order to avoid various distractions, Todd does not use a standard manipulation case, instead relying on a case involving God, who is all‐powerful, ‐knowing, and ‐benevolent. Todd imagines that God designs and creates a deterministic universe, with knowledge concerning every event that will occur, including the fact that Ernie will kill Jones for selfish reasons.^47^ Not only is it counterintuitive to claim that Ernie is blameworthy for killing Jones, it might be even more counterintuitive to claim that God has the standing to blame Ernie for it.

In order to account for this lack of standing, however, one must either explain (a) why Ernie is not blameworthy (since no‐one has the standing to blame the blameless) or (b) how God fails to meet some other condition on the standing to blame (e.g., perhaps blaming Ernie would be hypocritical, or his being involved in the creation of the universe makes him complicit in Ernie's murder). Todd argues that a denial of compatibilism—a form of (a)—is the best explanation for God's lack of standing. Todd begins with the assumption that the compatibilist's best hope for accounting for God's lack of standing is of form (b). He then proceeds to consider a variety of ways one might lack standing, arguing that none of them satisfactorily apply to God and concluding that his favored explanation is best.

There are, by now, various responses. King observes that there is something particularly problematic about *God*'s blaming Ernie, yet explanations of form (a)—including Todd's—fail to account for this, insofar as they apply to *anyone's* blame of Ernie (2015: 4). Some have suggested that switching from Diana to such a God—understanding him as bringing about the best of all possible worlds—brings with it complications; for example, if Ernie's action was necessary for bringing about the best of all possible worlds, then it may not be wrong, or perhaps the stipulated details of this God are controversial.^48^ Further, one might argue that, contra Todd, God is involved in the action in a way that undermines standing.^49^

This concludes Part 1 of this series on manipulation cases in free will and responsibility. For those interested in seeing how accounts of freedom and responsibility have been modified in light of discussions of manipulation cases and arguments, see Parts 2 and 3.^50^

## Conflicts of Interest

The authors declare no conflicts of interest.

## Data Availability

Data sharing not applicable as no datasets generated and/or analysed for this study.

## Endnotes

^1^
For some other useful surveys of the literature on manipulation cases and arguments, see (Capes 2023; Mickelson 2019a; Pereboom and McKenna 2022).

^2^
There are various versions of this case (Pereboom 1995, 2001, 112–3, 2014, 76–7, 2017 2021, 16), which plays an important role in Pereboom's version of the manipulation argument (see *n*. 23 below). For discussions of different versions of this case providing responses not covered below, or in the sequels, see (Baker 2006; Buckareff Forthcoming; Demetriou 2010; Fischer 2006, Chapter 12; Matheson 2016; McKenna 2017; Mele 2005; Pereboom 2017; Shabo 2010, 2012; Vihvelin 2013, 152).

^3^
Sometimes, a worry is raised concerning personal identity in such cases (or variations); for examples, see (Arpaly 2006, 116; Matheson 2014, 2019a; Vargas 2013, Chapter 9). For discussion, and arguments to the effect that personal identity can be preserved in such cases, see (Haji and Cuypers 2001, 222–3, 2008, 225, *n*. 4; Mele 1995, 175, *n*. 22, 2019, 75).

^4^
Pereboom's Case 2 (2014, 77) is also a case of original design. We return to his Four Case Argument below. It is often claimed that, though such cases are not cases of manipulation, this does not matter greatly for the debate. For some examples, see (Kearns 2012, 381, *n*. 2; Mele 2008, 285; Sartorio 2016, 160).

^5^
This makes *Ernie* a case of what Kane (Kane 1985, 35, 1996, 65) calls “covert non‐constraining (CNC) control,” since Ernie does what he wants, desires, and intends but is controlled by someone else so that he wants, desires, and intends only what the controller has planned. (Given how Kane characterizes CNC control, *Plum* and *Sweet Sally* are also instances).

^6^
There are variations on the premise. For instance, some further specify that Victim meets some compatibilist conditions, and resembles some of the earliest discussions of manipulation (and related forms of freedom‐undermining influence, such as coercion) in the literature on free will, where the case of manipulation was introduced as a counterexample to particular compatibilist accounts. See, for example (Slote 1980; Taylor 1963, 45–6), and see Double (1989) for a critical response to such efforts. See also King's (2013) presentation of the argument. Or, for instance, one might add a “because” clause, for example, “Because of the way his zygote was produced in his deterministic universe, Ernie is not a free agent and is not morally responsible for anything” (Mele 2006, 189). For some discussion, see (Kearns 2012; Todd 2013).

^7^
For some discussion, see (Mickelson 2019a; Sartorio 2016, 156–60).

^8^
For discussion, see (De Marco 2016; Mickelson 2015, 2021) and for more on whether the argument needs to be an argument to the best explanation, see (Mele 2006, 139–44, 2007, 203–6; Pereboom 2007, 168–171, 2014, 79–80, *n*. 3).

^9^
See, for instance (Sartorio 2016, 157–8).

^10^
For instance, one might use such cases (or arguments) to argue for particular soft‐line views (see Parts 2 and 3). Another version of the manipulation argument targets classical compatibilism in particular, where classical compatibilism is a form of compatibilism holding the view that freedom and responsibility requires the freedom to do otherwise. Cohen (2015) and Todd (2017) argue that certain manipulated agents lack the freedom to do otherwise and, given *No‐Diff,* conclude that classical compatibilism is false. The version of the manipulation argument that Whittle considers and responds to is along these lines as well (2021, 85–8, 187–90). And Miller (2014) and Sridharan (2016) use manipulation cases to argue against attributionist accounts of responsibility.

^11^
Haji and Cuypers posit a third condition on direct responsibility—an authenticity condition—intended to capture claims about agents' responsibility in manipulation cases (Haji 2008; Haji and Cuypers 2001, 2008).

^12^
McKenna credits Kane (1996, 67–8) for this terminology.

^13^
For a view that can constitute a soft‐line response to at least some cases, but fits neither of the descriptions below, see (Matheson 2019a).

^14^
For some examples, see (Barnes 2015; Deery and Nahmias 2017; Herdova 2021; Usher 2020; Waller 2014; Yaffe 2003).

^15^
For examples, see (De Marco 2023a; Fischer 2012, Chapter 12; Fischer and Ravizza 1998, Chapters 7–8; Haji 2008, Chapter 7; Haji and Cuypers 2008; McKenna 2016; Mele 1995, Chapter 9, 2006, Chapter 7, 2019).

^16^
Some suggest, for example, that there is no “one size fits all” approach to manipulation cases (Demetriou 2010, 601; McKenna 2008, 143; Mele 2019, 118).

^17^
For the general worry, see (Capes 2023, 148; Caruso 2021, 83–4; Caruso and Pereboom 2022, 29–30; McKenna 2008, 143–4; Pereboom and McKenna 2022, 182–3; Timpe 2012, 139).

^18^
For a similar view, see Watson (1999) and possibly Doris (2015, 30–33).

^19^
See, for example (Arpaly 2003, 166–7, 2006, 114–5; Kaiserman 2021, 3608–11; McKenna 2004, 183–4, 2016, 93; Vargas 2006, 365).

^20^
For discussion of such cases, see (Arpaly 2006, 111–12; Barnes 2015, 564; Brink 2021, 100; Caruso 2021, 85; De Marco 2023b; De Marco and Cyr Forthcoming; Deery and Nahmias 2017, 1272; Fischer 2011, 270, 2016, 50; Liu 2022; Mele 1995, 168–9, 2006, 141, 2019, 27, 31, 58, 94, 155, n.10; Mickelson 2019a, 171; Nelkin 2011, 57–9; Pereboom 1995, 25, 2001, 115, 2014, 79, 82; Sartorio 2016, 160–1).

^21^
A couple of points. First, just like soft‐line views of the bypassing variety faced issues with cases of original design, manipulator‐focused views face issues with cases of natural forces (see Part 2). Second, natural force cases tend to be variations on cases of mid‐life manipulation, not original design (though see (Sartorio 2016, 160–70)).

^22^
See also (Fischer 2011; Nelkin 2011, 51–60; Sartorio 2016, 160–70; Sekatskaya 2018).

^23^
Pereboom's version of the manipulation argument takes a different form than what we presented above. Having presented the four cases, and argued that there is no relevant difference between cases 1–4, Pereboom argues that the best explanation of the Plums' lack of responsibility for their decisions is that the decisions were causally determined by factors outside of the Plums' control (Fischer et al. 2024, 97–101; Pereboom 1995, 2001, Chapter 4, 2014, Chapter 4; Pereboom and McKenna 2022). For an earlier example of a best‐explanation version of the argument, see Blumenfeld (Blumenfeld 1988).

^24^
This is an ongoing discussion between Pereboom and McKenna (McKenna 2008, 2014 2017; Pereboom 2008, 2014, 91–99, 2017; Pereboom and McKenna 2022). See also Haas (2013a).

^25^
For the use of such cases in the context of manipulation cases, see (Arpaly 2003, 127–9, 2006, 109–116; Barnes 2016, 2325–6; Cyr 2020a: 2390; McKenna 2008, 156–7; Shaw 2014, 7–8). For responses to this use of cases, see (Haji and Cuypers 2008, 58–60; Mele 2006, 179–84, 2020, 3148). This is sometimes accompanied with other cases of instant agents (McKenna 2004, 180–1, 2016, 90–3). For discussion of such cases, see Part 3.

^26^
For some other discussions of what might be affecting our intuitions, see (Arpaly 2003, 162–168, 2006, 109–116; De Marco 2023b, 498–503; Matheson 2012; McKenna 2013, 107–111; Mele 2006, 138–144, 2018; Nelkin 2007, 2011, 51–60, 2013, 124–5; Pereboom 2018; Sartorio 2016, 160–70, 2018; Spitzley 2015; Vargas 2013, Chapter 9).

^27^
Despite being a hard‐line reply in denying *No‐FR*, such a response might also deny *No‐Diff*, depending on how the premise is stated. For other views that may fit this mold, see Part 2.

^28^
Brink considers a similar response (2021, 97–102).

^29^
For proponents of a view like this, see, for instance (Nelkin 2011; Whittle 2021; Wolf 1990).

^30^
Barnes offers similar considerations, wondering whether agents like Sally meet the *epistemic* condition on responsibility (2016, 2314–5). For some discussion, see Mele (2019: 63).

^31^
For similar cases, though lacking the stipulation that Victim becomes unconscious in the relevant alternative, see (McKenna 2016, 91, *n*. 16; Yaffe 2003, 339).

^32^
Deery and Nahmias (2023) claim that for manipulation arguments to work, the manipulator must be able to perfectly predict what the agent will do. Yet since even in *deterministic* worlds, an agent like Diana cannot perfectly predict what Ernie will do, such arguments fail. If one thinks that Victim lacks responsibility in these indeterministic variations, then one might think that manipulation arguments can work despite the Manipulators' lack of perfect prediction (though see their discussion on, 462–4).

^33^
See, for example (Clarke 2012; Haji and Cuypers 2001; Harrison 2008, 2010; King 2013; McKenna 2016, 87–8; Mele 2006, 52, 2013, 242; Pereboom 2001, 41–54, 2014, 47–8, 84, *n*. 7; van and Inwagen 1983, 132).

^34^
For a variation that involves good and bad alternatives, see (King 2013, 71).

^35^
For attempts to respond to this sort of case on behalf of the incompatibilist, see (Harrison 2008, 2010; Russell 2002, 603, *n*. 22, 2017, 164–5).

^36^
See references in *n*. 33 above.

^37^
See (Cyr 2016, 1089, n. 33; King 2013, 78; Mickelson 2019a, 167, 2019b; Tognazzini 2014, 368–9, *n*. 15).

^38^
Though see also (Tognazzini 2014, 362, *n*. 8).

^39^
For an argument that such a case will be difficult to produce (in virtue of something like Ekstrom's DP‐2 (Ekstrom 1999, 211)), see (De Marco 2018, 20–24).

^40^
For responses, see (Cyr 2016; Tognazzini 2014).

^41^
For responses, see (Pereboom 2014, 80; Todd 2013). Demetriou (2010, 600–5) offers a somewhat similar dilemma, targeted at Pereboom's Case 1 (*Plum* above). For another related dilemma targeted at cases of manipulation more generally, see (Yaffe 2003, 339–40).

^42^
For similar dilemmas, see (Schlosser 2015; Takasaki 2021). In offering this dilemma, Schlosser responds to Todd's response to Kearns, and offers a somewhat broader version that does not explicitly make reference to Vihvelin's toolhood explanation.

^43^
For a response to some of these dilemmas, see (Matheson 2018).

^44^
Todd constructs the argument around Pereboom's Case 2 but agrees with us that this case is relevantly similar to the case of Ernie (2011, 128, *n*. 1).

^45^
Khoury (2014) responds to Tierney and offers an argument *for* compatibilism that is parallel to MMA, arguing that, since both are equally powerful, MMA's threat is defused. See Tierney (2014) for a reply.

^46^
See also (Todd 2018). In a paper pre‐dating Todd's, Russell offers a related version of the hard‐line response to manipulation cases, arguing, roughly, that though Victim is responsible, Manipulator lacks the standing to blame them (2010, 2017, Chapter 9).

^47^
For another instance of this sort of case to argue for incompatibilism (though without appealing to standing), see (Rosen 2002), and for a reply (Wallace 2002).

^48^
(King 2015; Matheson 2019b; McKenna 2018)

^49^
(Capes forthcoming; King 2015; Matheson 2019b; McKenna 2018; Russell 2010).

^50^
Unfortunately, though we have tried to cover as much ground as possible, there are more papers we have had to leave out of all three parts. Jeppsson (2020) offers a hard‐line response to the four‐case argument, making use of insights from Strawson's (1962) seminal paper (see Seybold (2022) for a reply). Latham and Tierney (2022) also make use of Strawson's work in responding to the argument, and have run empirical studies to test their view (Latham, Tierney, and Varga 2023; Latham and Tierney 2023). Amaya (2023) argues that theories of vagueness can help to illuminate the dialectic around manipulation arguments. Collins and de Haan (2024) consider cases of manipulation at the level of group agents. Huoranszki (2021) offers a response to the zygote argument. Matheson considers cases of self‐manipulation, and their implication for theories of responsibility (Matheson 2023). Torek (2022) argues that manipulation arguments mis‐represent determinism, and once we consider scientific theories of determinism, there is a clear difference between Victims and their merely determined counterparts. Alm (2015) argues that a relevant difference between manipulated and ordinary determined agents is that the former have (while the latter lack) a reason to resent being manipulated, which Alm also takes to be sufficient for lacking responsibility. Haas (2013b) argues that Pereboom and McKenna are using different notions of desert when debating Pereboom's four‐case argument (see *n*. 24 above) and suggests that progress can be made in this debate by attending to the various notions of desert.
